# Influence of *APOE* and *RNF219* on Behavioral and Cognitive Features of Female Patients Affected by Mild Cognitive Impairment or Alzheimer’s Disease

**DOI:** 10.3389/fnagi.2018.00092

**Published:** 2018-04-13

**Authors:** Alessandra Mosca, Samantha Sperduti, Viorela Pop, Domenico Ciavardelli, Alberto Granzotto, Miriam Punzi, Liborio Stuppia, Valentina Gatta, Francesca Assogna, Nerisa Banaj, Fabrizio Piras, Federica Piras, Carlo Caltagirone, Gianfranco Spalletta, Stefano L. Sensi

**Affiliations:** ^1^Department of Neuroscience, Imaging, and Clinical Science, Università degli Studi G. d’Annunzio Chieti e Pescara, Chieti, Italy; ^2^Department of Neuroscience, Psychology, Drug Area and Child Health, University of Florence, Florence, Italy; ^3^Molecular Neurology Unit, Center of Excellence on Aging and Translational Medicine, Università degli Studi G. d’Annunzio Chieti e Pescara, Chieti, Italy; ^4^Department of Psychological, Health and Territorial Sciences, School of Medicine and Health Sciences, Università degli Studi G. d’Annunzio Chieti e Pescara, Chieti, Italy; ^5^Department of Neurology and Pharmacology, Institute for Memory Impairments and Neurological Disorders, University of California, Irvine, Irvine, CA, United States; ^6^School of Human and Social Science, Kore University of Enna, Enna, Italy; ^7^Department of Clinical and Behavioral Neurology, Neuropsychiatry Laboratory, IRCCS Santa Lucia Foundation, Rome, Italy

**Keywords:** dementia, mild cognitive impairment (MCI), Alzheimer disease (AD), *APOE*, *RNF219*, genotype

## Abstract

The risk for Alzheimer’s disease (AD) is associated with the presence of the 𝜀4 allele of Apolipoprotein E (*APOE*) gene and, recently, with a novel genetic variant of the *RNF219* gene. This study aimed at evaluating interactions between *APOE*-𝜀4 and *RNF219*/G variants in the modulation of behavioral and cognitive features of two cohorts of patients suffering from mild cognitive impairment (MCI) or AD. We enrolled a total of 173 female MCI or AD patients (83 MCI; 90 AD). Subjects were screened with a comprehensive set of neuropsychological evaluations and genotyped for the *APOE* and *RNF219* polymorphic variants. Analysis of covariance was performed to assess the main and interaction effects of *APOE* and *RNF219* genotypes on the cognitive and behavioral scores. The analysis revealed that the simultaneous presence of *APOE*-𝜀4 and *RNF219*/G variants results in significant effects on specific neuropsychiatric scores in MCI and AD patients. In MCI patients, *RNF219* and *APOE* variants worked together to impact the levels of anxiety negatively. Similarly, in AD patients, the *RNF219* variants were found to be associated with increased anxiety levels. Our data indicate a novel synergistic activity *APOE* and *RNF219* in the modulation of behavioral traits of female MCI and AD patients.

## Introduction

Alzheimer’s disease is a complex syndrome characterized by a pleiotropic array of cognitive and behavioral symptoms (Selkoe, 2011). AD is mainly driven by the intraneuronal accumulation of β-amyloid, the extracellular formation of amyloid plaques and the appearance of intracellular neurofibrillary tangles composed of phosphorylated tau proteins. More recent lines of evidence support the idea that imbalance of β-amyloid production and clearance, along with phosphorylated tau and the interplay with other co-morbidity factors (metabolic, vascular, and inflammatory) work synergistically on a permissive condition represented by the aging brain to promote AD (Herrup, 2010; Corona et al., 2011; Selkoe and Hardy, 2016). Genetic and environmental factors also affect the onset and progression of the disease (Tanzi, 2012; Raskin et al., 2015).

Genome-wide-association studies have identified and confirmed more than 20 genetic variants associated with higher susceptibility to develop Late-Onset Alzheimer Disease (LOAD) of the sporadic type (Winblad et al., 2015). Among these, the 𝜀4 allele is a specific variant of the Apolipoprotein E gene (*APOE*-𝜀4) and a significant risk factor for AD (Saunders et al., 1993; Bertram et al., 2007). The physio-pathological function of *APOE* is complex (Ossenkoppele et al., 2013; Tai et al., 2015) as the gene can interfere in many ways with the pillars of the disease (Ohm et al., 1999; Tanzi, 2012). As an integral part of cellular membranes, *APOE*-𝜀4 can influence the amyloidogenic processing of the APP and impair its clearance from the brain (Selkoe and Hardy, 2016). *APOE*-𝜀4 can also promote tau phosphorylation (Zhou et al., 2016) and affect metabolic and vascular factors such as hypertension, diabetes mellitus, as well as the metabolic syndrome. All these factors synergistically modulate the AD onset and progression (Duron and Hanon, 2008; Toledo et al., 2013). For instance, these factors target the physiological functioning of the neurovascular unit and the BBB integrity. Interestingly, *APOE*-𝜀4 has been recently shown to converge on this critical step by affecting the operation of the neurovascular unit and promoting the breakdown of proteins responsible for the BBB integrity (Montagne et al., 2015; Zhao et al., 2015). However, despite the growing body of evidence on the *APOE*-related pathogenic mechanisms, a definitive molecular roadmap on the 𝜀4 haplotype targets remains elusive.

Recent data also indicate that a genetic variant of the *RNF219* gene may increase the risk for the AD (Rhinn et al., 2013). The rs2248663 A>G (*RNF219*/G) polymorphism of the *RNF219* gene encoding for a member of the RNF family, has been associated with earlier onset of AD when working in synergy with the *APOE*-𝜀4 (Rhinn et al., 2013). This accelerating effect is not present in non-𝜀4 and *RNF219*/A carriers, thereby indicating that the two genes may work on common pathogenic pathways. In the study, we set out to integrate with new evidence the original *RNF219* findings (Rhinn et al., 2013) and evaluated whether *APOE*-𝜀4 and *RNF219*/G work in synergy or independently to affect the behavioral or cognitive features of patients affected by mild cognitive impairment (MCI) or AD. To that aim, we analyzed a comprehensive set of behavioral and cognitive profiles in two cohorts of female MCI or AD patients that included carriers or non-carriers of *APOE*-𝜀4 and *RNF219*/G.

## Materials and Methods

### Study Population

The study was approved by the Institutional and Ethics Committee of the I.R.C.C.S. Santa Lucia-Rome. All procedures were conducted in accordance with principles expressed in the Helsinki Declaration. We recruited 173 total volunteers (mean age ± standard deviation = 74 ± 7) including 83 MCI and 90 AD patients. All included subjects signed an informed consent form before enrolment. Clinical evaluations were conducted by trained psychologists and AD specialists (neurologists and psychiatrists).

### Neuropsychological Assessment

Subjects were assessed with the following neuropsychological tests: MMSE, RAVL, Phonemic Verbal Fluency, CPMs, complex figure of Rey, Stroop test, and NPI. The main functional capacity was assessed by daily non-instrumental (ADL) (Wallace et al., 2007) and instrumental activities (IADL) (Lawton and Brody, 1969).

Mini Mental State Examination defines the global level of cognitive deterioration on a scale of 0–30 and targets general mental abilities, memory, attention, and language. A Score greater than or equal to 24 indicates the absence of cognitive deficits, scores ≤ 9 indicate the presence of severe cognitive deficits, scores between 10 and 18 indicate moderate cognitive deficits, and scores between 19 and 23 indicate mild cognitive deficits (Folstein et al., 1975). RAVL allows a quantitative assessment of the ability of immediate and delayed recall (Snyder and Harrison, 1997). The CPMs measure fluid intelligence (Basso et al., 1987). The complex figure of Rey is a visual-perceptual test that investigates the complex perceptual organization and long-term visual memory (Shin et al., 2006). The Stroop test examines aspects of attention and executive functions (Tremblay et al., 2016). The NPI was developed to provide a way to assess neuropsychiatric symptoms and psychopathology of patients with AD and other neurodegenerative disorders (Cummings et al., 1994). The NPI has been therefore employed to characterize neuropsychiatric profiles and is a structured interview that evaluates the following 12 behavioral domains: delusions, hallucinations, agitation, dysphoria, anxiety, apathy, irritability, euphoria, disinhibition, aberrant motor behavior, night-time behavioral disturbance, eating disorders, and weight changes.

### DNA Extraction

For gene variants analysis, genomic DNA was isolated from blood samples by the PureLink Genomic DNA Mini Kit (Life Technologies, Carlsbad, CA, United States), quantified by an Agilent 8453 Spectrophotometer (Agilent, Santa Clara, CA, United States) and stored at -20°C.

### APOE Genotyping

*APOE* genotyping was performed by direct sequencing. PCR amplification of the region containing the rs429358 and rs7412 sites that determine the 𝜀2, 𝜀3, or 𝜀4 variants of the gene was carried out using the primers pair Forward: 5′-TAAGCTTGGCACGGCTGTCCAAGGA-3′ and Reverse: 5′-ACAGAATTCGCCCCGGCCTGGTACAC-3′, resulting in a 244 bp fragment (Hixson and Vernier, 1990). Purified PCR products were sequenced by the BigDye Terminator v3.1 Cycle Sequencing Kit (Life Technologies, Carlsbad, CA, United States) according to the manufacturer protocol. Sequence products were then separated on an ABI 3130xl automatic sequencer (Applied Biosystems, Paisley, United Kingdom) and analyzed using Sequencing Analysis Software (Applied Biosystems, Paisley, United Kingdom).

### RNF219 Genotyping

*RNF219* genotyping was carried out using High-Resolution Melting Analysis in 48-well plates on a StepOne^TM^ Real-Time PCR System run by StepOne Software v2.2.2 (Applied Biosystems, Paisley, United Kingdom) and analyzed with High-Resolution Melt Software v3.0.1 (Life Technologies, Carlsbad, CA, United States). We amplified a 103 bp fragment using the following primers pair: Forward: 5′-GGAAAAAGACAATGCAGGAAT-3′; Reverse: 5′-TTTTACCAAGGGCAACATTTC-3′. The PCR reaction, containing 20 ng of genomic DNA and the MeltDoctor HRM Master Mix (Applied Biosystems), according to the manufacturer protocols, was run as follow: enzyme activation at 95°C for 10 min; 40 cycles of denaturation and extension at 95°C for 15 s and 60°C for 1 min; melt curve with a denaturation at 95°C for 10 s, annealing at 60°C for 1 min, high resolution melting from 60 to 95°C with a ramp rate of 0.3% and final re-annealing at 60° C for 15 s. Fluorescence signals were measured during the amplification and melting steps.

### Statistical Analysis

*APOE* and *RNF219* genotypic and allelic frequencies of female MCI and AD patients were calculated as previously described (Wigginton et al., 2005). For statistical analysis, we separated the MCI and AD cohorts in carriers and non-carriers of the two allelic variants 𝜀4 and G. Allele frequencies of both *APOE* and *RNF219* polymorphisms were assessed for Hardy–Weinberg equilibrium (HWE) using a chi-square (χ^2^) test with significance set at *p* < 0.05 (Wigginton et al., 2005).

One-way analysis of variance (ANOVA) followed by Fisher least significant difference (LSD) *post hoc* test was performed to investigate the significance of differences between age, education levels, MMSE corrected for age and education levels, the reported (by the patient or caregivers) age of appearance of the first symptom for MCI subjects, and age of onset for AD patients. Levene test was performed for assessment of homoscedasticity of the groups. Kruskal–Wallis test followed by multiple comparison of mean ranks was performed when the variances between groups were non-homogeneous.

Analysis of covariance (ANCOVA) was performed using a general linear model (GLM) approach and controlling for age and education level. *APOE* and *RNF219* genotypes were the independent factors, and the neuropsychological scores were the dependent variables. The main and interaction effects of the APOE and RNF219 genotypes were evaluated. The employed ANCOVA model is as follow: Y_i_ = β_0_ + β_1_ (age_i_) + β_2_ (education level_i_) + β_3_ (*APOE* genotype_i_) + β_4_ (*RNF219* genotype_i_) + β_5_ (*APOE* genotype_i_ × *RNF219* genotype_i_) + ε_i_, where Y_i_ indicates the specific i^th^ neuropsychological score, β_0_ is the intercept, and ε_i_ is the error term associated with the model. In the case of ordinal variables or when the assumption of the homogeneity of the variance was rejected by the Levene test, the ART procedure was applied (Wobbrock et al., 2011; Feys, 2016). Multiple comparisons were performed using Fisher LSD *post hoc* test.

In all cases, *p*-values were corrected for multiple comparisons using the Benjamini–Hochberg correction at a false discovery rate (FDR) of 5%. *p*-Values < 0.050 were considered statistically significant. Statistical analysis was performed using Statistica 6.0 software (Statsoft, Tulsa, OK, United States).

## Results

### Demographic and Clinical Features of MCI and AD Cohorts

The demographic and clinical characteristic of the study groups in the MCI or AD cohorts are shown in **Table 1**. The study subgroups were matched for age, education levels, and MMSE scores as well as for the reported age of the first symptoms (in the case of MCI subjects) or age of onset (in the case of AD patients).

**Table 1:** Demographic and clinical features of the study groups.

Characteristic	MCI				Levene test, *p*	One-way ANOVA or Kruskal–Wallis test, *p*
	*APOE*-*𝜀4* carrier		*APOE*-*𝜀4* non-carrier			
	*G carrier*	*G non-carrier*	*G carrier*	*G non-carrier*		
Number of participants	5	31	10	37		
Age, years; mean (SD)	72 (7)	71 (7)	69 (7)	73 (6)	0.80	0.34
Education level, mean (SD)	9 (5)	9 (5)	6 (3)	7 (3)	0.060	0.084
Reported age of first symptom, years; mean (SD)	70 (8)	68 (7)	67 (7)	71 (6)	0.76	0.39
MMSE, mean (SD)	25.5 (0.9)	25 (2)	27 (1)	26 (2)	0.069	0.22

**Characteristic**	**AD**				**Levene test, *p***	**One-way ANOVA or Kruskal–Wallis test, *p***
	***APOE*-*𝜀4* carrier**		***APOE*-*𝜀4* non-carrier**			
	***G carrier***	***G non-carrier***	***G carrier***	***G non-carrier***		

Number of participants	8	26	10	46		
Age, years; mean (SD)	79 (5)	74 (8)	79 (9)	77 (8)	0.41	0.19
Education level (years in school), mean (SD)	7 (4)	9 (5)	6 (4)	7 (3)	0.053	0.091
Age of onset, years; mean (SD)	77 (5)	72 (7)	77 (9)	75 (8)	0.52	0.18
MMSE, mean (SD)	21 (5)	20 (5)	17 (7)	20 (4)	0.41	0.21

*Data are depicted as means and standard deviations (SD). Statistical analysis was performed using one-way analysis of variance (ANOVA) followed by Fisher least significant difference *post hoc* test or Kruskal–Wallis test followed by multiple comparison of mean ranks. Levene test was performed for assessment of homoscedasticity of the groups. False discovery rate (FDR) corrected *p-*values < 0.050 are shown in bold. MCI, patients with mild cognitive impairment; AD, patients with Alzheimer’s disease; MMSE, mini-mental state examination score corrected for age and education levels; G carrier, RNF219/G polymorphism carrier, G non-carrier, RNF219/G polymorphism non-carrier; APOE genotype, APOE-𝜀4 genotype; 𝜀4 carrier, APOE-𝜀4 genotype carrier; 𝜀4 non-carrier, APOE-𝜀4 genotype non-carrier.*

### Distribution of *APOE* and *RNF219* Genotypes in the MCI and AD Cohorts

The distribution of *APOE* and *RNF219* genotypes and relative frequencies in MCI and AD patients are shown in **Table 2**. Genotypes were in the Hardy–Weinberg equilibrium in MCI (*APOE p* = 0.064; *RNF219 p* = 0.36) and AD (*APOE p* = 0.64; *RNF219 p* = 0.29) patients.

**Table 2:** Allele and genotype frequencies of APOE and RNF219 polymorphisms in the MCI and AD groups.

	*MCI* (*n* = 83)			*AD* (*n* = 90)		
		*n*	*Frequency* (%)		*n*	*Frequency* (%)
*APOE 𝜀2/𝜀3/𝜀4 genotypes*	𝜀2/𝜀2	0	0	𝜀2/𝜀2	0	0
	𝜀2/𝜀3	4	4.8	𝜀2/𝜀3	2	2.2
	𝜀3/𝜀3	43	51.8	𝜀3/𝜀3	54	60
	𝜀3/𝜀4	34	41	𝜀3/𝜀4	31	34.4
	𝜀4/𝜀4	0	0	𝜀4/𝜀4	2	2.2
	𝜀2/𝜀4	2	2.4	𝜀2/𝜀4	1	1.2
*APOE 𝜀2/𝜀3/𝜀4 alleles*	𝜀2	6	3	𝜀2	3	2
	𝜀3	124	75	𝜀3	141	78
	𝜀4	36	22	𝜀4	36	20
*RNF219 rs2248663 A > G genotypes*	A/A	68	81.9	A/A	72	80
	A/G	15	18.1	A/G	18	20
	G/G	0	0	G/G	0	0
*RNF219 rs2248663 A > G alleles*	A	151	91	A	162	90
	G	15	9	G	18	10

### Effects of the *APOE* and *RNF219* Genotypes on Behavioral Features of MCI Subjects

Our study revealed that, in MCI subjects, the anxiety-related NPI score depends on the interaction between *APOE* and *RNF219* genotypes (*p* = 0.003) (**Supplementary Table S1**). The *APOE* genotype alone showed a trend toward significant effect on the same NPI score (*p* = 0.074) (**Supplementary Table S1**). In contrast, we did not find significant effects of age or education on the anxiety trait (*p* = 0.063 and 0.16, respectively).

**FIGURE 1:**
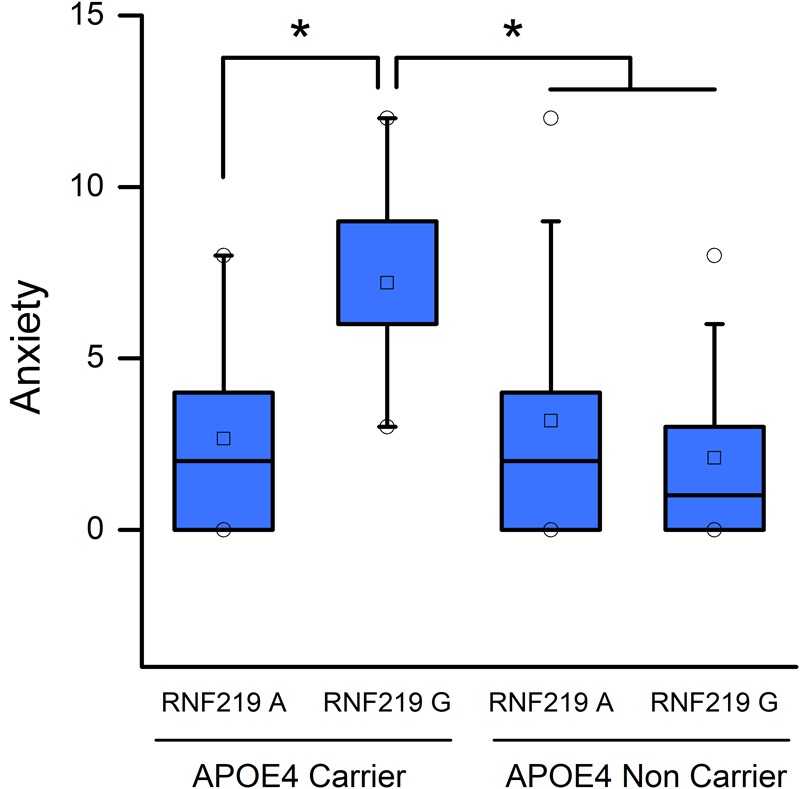
Apolipoprotein E (APOE) and RNF219 interaction in the modulation of anxiety of MCI subjects. Box plots show a comparison of anxiety scores and statistical differences set at *p* < 0.05. Squares depict the mean values. The central horizontal bars represent the median values. The lower and the upper limits of the box represent the first and the third quartiles, respectively. Circles represent the minimum and maximum values of anxiety scores. Note that 𝜀4/G carriers show increased anxiety-related NPI scores compared to 𝜀4/A carriers (*p* = 0.009), non-𝜀4/A carriers (*p* = 0.017), and non-𝜀4/G carriers (*p* = 0.009). ^∗^Indicate statistically significant differences.

*Post hoc* multiple comparisons showed that MCI 𝜀4/G carriers displayed increased levels of anxiety compared to other groups of patients. In fact, MCI patients carrying the 𝜀4/G alleles show higher levels of anxiety [median (interquartile range): 6 (6–9)] compared to MCI 𝜀4/A carriers [median (interquartile range): 2 (0–4); *p* = 0.009], non-𝜀4/A carriers [median (interquartile range): 2 (0–4); *p* = 0.017] and non-𝜀4/G carriers [median (interquartile range): 1 (0–2.75); *p* = 0.009; **Figure 1**].

In contrast, we did not find significant main and/or interaction effects of *APOE* and *RNF219* variants on the other neuropsychological scores (**Supplementary Table S1**).

### Effects of the *APOE* and *RNF219* Genotypes on Behavioral Features of AD Patients

**FIGURE 2:**
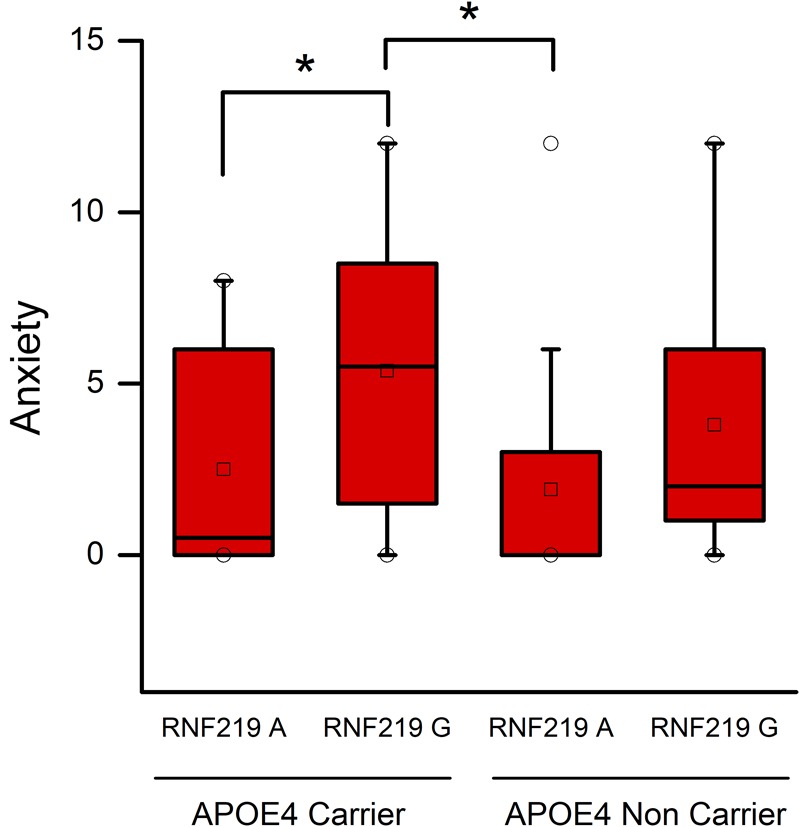
Apolipoprotein E and RNF219 interaction in the modulation of anxiety of AD patients. Box plots show a comparison of anxiety NPI scores and statistical differences set at *p* < 0.05. Squares depict the mean values. The central horizontal bars represent the median values. The lower and the upper limits of the box represent the first and the third quartiles, respectively. Circles represent the minimum and maximum values of anxiety scores. Note that 𝜀4/G carriers show higher anxiety-related NPI scores compared to 𝜀4/A (*p* = 0.041) and non-𝜀4/A carriers (*p* = 0.030). ^∗^Indicate statistically significant differences.

In the case of AD patients, we found that *RNF219* variants had significant effects on anxiety-related NPI scores (*p* = 0.015). Similarly to the MCI group, in the AD cohort, we found that 𝜀4/G carriers show higher anxiety-related NPI scores [median (interquartile range): 5.50 (1.75–8.25)] compared to 𝜀4/A [median (interquartile range): 0.5 (0–5.5); *p* = 0.041; **Figure 2**] and non-𝜀4/A carriers [median (interquartile range): 0 (0–2.75); *p* = 0.030; **Figure 2**].

As for MCI subjects, we did not find any significant differences in other neuropsychological scores of the AD cohort (**Supplementary Table S2**).

## Discussion

In the study we explored whether *APOE*-𝜀4 and *RNF219*/G work in synergy or independently to affect the behavioral or cognitive features of MCI and AD patients (Rhinn et al., 2013).

In a preliminary phase of the study, we attempted to evaluate the synergistic effects of *APOE* and *RNF219* variants on behavioral and cognitive traits of male and female MCI or AD patients. However, after genotyping, we found that the sample size was too small to evaluate the effects of RNF genotype in males. Therefore, the study was redirected to investigate the impact of *APOE*-ε4 and *RNF219*/G only in female patients. We acknowledge that this is a limitation of our study and further studies will need to address effects on male patients.

In the study, we found that the *RNF219*/G variant, in synergy with the *APOE*-ε4 allele, amplifies the anxiety-related NPI scores. These scores are higher in *APOE*-𝜀4 and *RNF219*/G carriers of the MCI or AD cohorts (**Figures 1, 2**).

Anxiety disorders are common late-life psychiatric features and have been associated with lower cognitive performance in older adults (Beaudreau and O’Hara, 2008). Several lines of evidence support the modifying effect of the *APOE*-𝜀4 status on the AD neuropsychiatric symptoms (Ungar et al., 2014). Reports indicate that anxiety and other behavioral symptoms are more prominent and severe in the population of female AD patients who are *APOE*-𝜀4 carriers (Steinberg et al., 2006; Xing et al., 2015), thereby supporting the notion of a relationship between the interaction of *APOE*-𝜀4 and gender in the phenotypical shaping of the AD-related behavioral features. The precise biological underpinning of the phenomenon is difficult to be identified. One possibility relies on the role played by estrogens in the disease progression of female patients. These hormones affect the synaptic plasticity of the AD brain as well as shape the response to AD-related pathology (Yaffe et al., 2000; Carroll and Rosario, 2012; Kang and Grodstein, 2012; Kramár et al., 2013). Hormonal changes can act on neurotrophic mechanisms and be responsible for behavioral symptoms. For instance, in females, decreased peri-menopausal levels of estrogens have been suggested to favor the onset and progression of dementia-related depression and anxiety (Aloysi et al., 2006). These estrogen-related effects can amplify the activity of *APOE*. In fact, it is well-known that *APOE*-𝜀4 allele acts as a negative modulator of neuropsychiatric features in AD patients (Spalletta et al., 2006; Steinberg et al., 2006; Panza et al., 2012). Moreover, levels of estradiol are known to be influenced by the expression of the *APOE*-𝜀4 allele and promote a worsening of neuropsychiatric symptoms in female *APOE*-𝜀4 carriers (Xing et al., 2012). Surprisingly, we did not find significant effects of the *APOE*-𝜀4 allele on neuropsychological features such as apathy, aggressiveness, and depression. These symptoms have been previously shown in MCI or AD patients (Panza et al., 2011). A possible explanation of these divergent results may depend on the fact that our study has evaluated only female subjects while others have investigated mixed groups that included female and male patients.

The neurobiological effects of *RNF219* remain most unexplored. *RNF219* belongs to a family of proteins pleiotropically involved in many cellular functions. Some RNF proteins have been shown to modulate myelin formation (Hoshikawa et al., 2008) and the stability of GABAergic synapses (Jin et al., 2014). These proteins interfere with the activation of the ubiquitin system (Joazeiro and Weissman, 2000), a crucial mechanism for neuronal demise (Zheng et al., 2014). A role for selected RNF proteins has also been proposed in neurodegenerative processes (Pranski et al., 2013; Matz et al., 2014). In that regard, several genetic variants at the *RNF219* locus have been associated with the presence of cognitive deficits, brain atrophy and lipid deregulation (Barber et al., 2010; Cirulli et al., 2010; Furney et al., 2011). Of note, the *RNF219*/G variant has been recently associated with an earlier onset of AD (Rhinn et al., 2013).

Interestingly, recent studies in MCI patients have reported a positive relationship between the presence of high levels of anxiety and the likelihood of conversion to AD. Although the issue remains controversial (Devier et al., 2009; Breitve et al., 2016), it has been shown that anxiety is associated with the earlier conversion to AD (Gallagher et al., 2011; Mah et al., 2015). Therefore, our findings allow the speculation of a potential correlation between anxiety, RNF219/G, *APOE*-𝜀4 and the conversion to AD.

In our study, we did not find any significant correlation between the anxiety levels and an earlier onset age for the first cognitive symptoms for MCI subjects or AD clinical signs (data not shown). *RNF219*/G has been shown to favor an earlier presentation of the disease in AD patients who are carriers of the polymorphism. The discrepancy with our study may be related to the small sample size of our female study groups and/or a gender effect. Our findings instead show the presence of higher anxiety levels in patients who are carrying *APOE*-𝜀4 and *RNF219*/G. This result may support the idea of a synergistic effect of these alleles on the behavioral alteration of the disease. Future studies are needed to clarify whether and how *RNF219*/G plays in synergy with the gender and *APOE*-𝜀4 status to affect the neurodegenerative processes underlying dementia.

## Author Contributions

SLS and GS: designed the study. AM and SS: performed the experiments. AM, SS, DC, AG, MP, FaP, NB, FeP, FA, VP, LS, CC, GS, and VG: analyzed the data and interpreted the results. SS, AM, and SLS: wrote the paper. All authors approved the final version of the manuscript.

## Conflict of Interest Statement

The authors declare that the research was conducted in the absence of any commercial or financial relationships that could be construed as a potential conflict of interest.

## Supplementary Material

TABLE S1 Summary data and Analysis of Covariance (ANCOVA) of neuropsychological scores in MCI subjects. ANCOVA was performed in order to evaluate the main and interaction effects of APOE and RNF219 genotype controlling for age and education level. Significant *p*-values, corrected for multiple comparisons using the Benjamini–Hochberg correction at a false discovery rate (FDR) of 5%, are shown in bold.

TABLE S2 Summary data and analysis of covariance (ANCOVA) of neuropsychological scores in AD patients. ANCOVA was performed in order to evaluate the main and interaction effects of APOE and RNF219 genotype controlling for age and education level. Significant *p*-values, corrected for multiple comparisons using the Benjamini–Hochberg correction at a false discovery rate (FDR) of 5%, are shown in bold.

## Abbreviations

AD: Alzheimer disease
APOE: apolipoprotein E
APP: amyloid precursor protein
ART: Aligned Rank Transformation
BBB: blood–brain barrier
bp: base pair
CPM: Colored Progressive Matrices
GWASs: genome-wide-association studies
MCI: mild cognitive impairment
MMSE: Mini Mental State Examination
NPI: Neuropsychiatric Inventory
PCR: polymerase chain reaction
RAVL: Rey Auditory Verbal Learning
RNF: Ring Finger Protein
